# A framework for extending co-creative communication models to sustainability research

**DOI:** 10.3389/frai.2024.1236310

**Published:** 2024-08-05

**Authors:** Guanhong Li, Xiaoyun Guo

**Affiliations:** ^1^Department of Global Tourism, Faculty of Global Engagement, Kyoto University of Foreign Studies, Kyoto, Japan; ^2^Independent Researcher, Kyoto, Japan

**Keywords:** co-creative communication, innovation, social systems theory, sustainability, Sustainable Development Goals, transition systems, uncertainty

## Abstract

The UN Sustainable Development Goals (SDGs) present a challenge due to their potential for conflicting objectives, which hinders their effective implementation. In order to address the complexity of sustainability issues, a framework capable of capturing the specificity of diverse sustainability issues while offering a common methodology applicable across contexts is required. Co-creative communication can be regarded as a key source of uncertainty within functional systems, as it can be instrumental in realizing and sustaining sustainability. In this regard, the studies in Constructive approaches to Co-creative Communication (CCC), particularly those employing artificial intelligence (AI) methodologies such as computational social science and innovation studies, hold significant value for both theoretical and applied sustainability research. However, existing CCC frameworks cannot be directly applied to sustainability research. This work bridges this gap by proposing a framework that outlines a general approach to establishing formalized definitions of sustainability from the lens of communication. This approach enables the direct application of CCC models to sustainability studies. The framework is based on systems theory and the methodologies of artificial intelligence, including computational/symbolic modeling and formal methods. This framework emphasizes the social function of co-creative communication and the interaction between the innovation process and the sustainability of the system. It can be concluded that the application of our framework enables the achievements of CCC to be directly applied to sustainability research. Researchers from different disciplines are therefore able to establish their own specific definitions of sustainability, which are tailored to their particular concerns. Our framework lays the groundwork for future sustainability studies that employs CCC, facilitating the integration of CCC insights into sustainability research and application. The outcomes of computational creativity research based on AI technologies, such as distributed artificial intelligence and self-organizing networks, can deepen the understanding of sustainability mechanisms and drive their practical applications. Furthermore, the functional role of co-creative communication in societal sustainability proposed in this work offers a novel perspective for future discussions on the evolutionary adaptation of co-creative communication.

## 1 Introduction

Sustainability has received worldwide attention as a fundamental challenge for human society. The most widely used definition of sustainability or sustainable development is probably that proposed in the 1987 report *Our Common Future* (also known as *Bundtland Report*) of the World Commission on Environment and Development (WCED) established by the United Nations (UN) (World Commission on Environment and Development, 1987). This report defined sustainability as the problem of meeting the needs of the present without compromising the ability of future generations to meet their own needs. The report is considered important because it succeeded in bringing sustainability to the public attention (Mitlin, 1992). The researchers noted that the report was actually based on several earlier studies, including the 1972 project *The Limits To Growth (LTG)* (Mitlin, 1992; Mensah, 2019). In the LTG project, the authors used a constructive approach and reported that their model predicted that the limits to human growth would be reached before the year 2072 (Meadows et al., 2019).

Although earlier studies on sustainability were more concerned with the relationship between economic development and natural resources, more recent studies have seen sustainability as a much broader issue. Researchers have argued that sustainability can be seen as an issue focusing on equity within and between generations (Mensah, 2019). Specifically, it is argued that there are three main sustainability issues: economic growth, environmental protection, and social equity, which are seen as the three “pillars” of the concept of sustainability. In fact, the UN 2030 Agenda lists 17 Sustainable Development Goals (SDGs), which cover a wide range of economic, environmental and social issues (UN General Assembly, 2015). However, there is evidence that the SDGs have had a limited impact on our policies (Biermann et al., 2022). A major reason may be the inherent incompatibility between the SDGs (Spaiser et al., 2017), which involve trade-offs and competing interests and require compromises between different goals (Mensah, 2019; Raimbault and Pumain, 2022).

From the above, two main problems can be identified with the current definition of sustainability. First, due to inherent incompatibilities, sustainability cannot be addressed by merely addressing specific issues in their respective domains. Second, defining sustainability as a whole-of-society issue requires the collaboration and integration of different social sectors across society, which is difficult in practice. Therefore, this study focuses on the approach to define sustainability in different contexts. We expect that the new approach can be generally applied to different domains without relying on cross-domain collaboration and integration.

The difficulties in achieving sustainability can be understood through Luhmann's Social Systems Theory (SST) (Luhmann, 1995). Luhmann's theory views society as an autopoietic system in which communication is reproduced. According to this theory, modern society can be functionally divided into different subsystems, such as economy, law, and politics. These functional systems are connected to each other through structural coupling (Luhmann, 1995; Iba, 2016). Therefore, it can be argued that the high degree of differentiation of social functions makes it difficult to address global ecological problems, which would require a society-wide collaboration that is unlikely to be successful (Bergthaller, 2018).

For a modern society, it is difficult to solve global sustainability problems at the level of the entire society. Instead, we believe that a practical solution to sustainability is to consider sustainability within individual functional systems in society. This position is consistent with Luhmann's view of ecological problems. In this paper, the term *social system* is used to refer to a functionally independent system in modern society.

Insufficient uncertainty emerges as a core factor underlying sustainability issues when viewed through the lens of social systems. Sustainability of social systems is closely related to Ivan Illich's concept of counterproductivity, particularly exemplified in contemporary medical systems. Russell (2019) notes that beyond a certain institutional scale or intensity, the proliferation of medical interventions paradoxically exacerbates rather than alleviates health problems. This issue can be illustrated from the lens of Luhmann's SST. Within SST, the maintenance of system functionality relies on communication, which, in turn, requires the media to mitigate uncertainty (Naruse and Iba, 2008; Iba, 2016). Consequently, system functionality is achieved through uncertainty reduction. When the reducible uncertainty falls short, it manifests as a problem of excessive communication. At this point, the system functionality becomes unsustainable, leading to counterproductive outcomes. Thus, from the perspective of independent functional systems, the inadequacy of uncertainty, often precipitated by overcommunication, emerges as a pivotal reason for the contemporary challenge of sustainability.

Luhmann's SST faces limitations when considering the impact of technological development, particularly in terms of communication media. Social systems, in their pursuit of functional realization, naturally seek to enhance communication efficiency. Technological advances, particularly the development and application of platforms such as social networking services (SNS), virtual reality (VR), and generative AI, have profoundly altered contemporary communication environments, resulting in extensive social impacts. However, Luhmann did not foresee the implications of such technological developments (Gerim, 2017). On one hand, AI technologies can be harnessed to address societal issues, such as supporting collective decision-making (Raikov, 2018). On the other hand, they also have the potential for adverse effects, as evidenced by the emergence of generative AI (Lamb and Brown, 2023). We contend that it is the rapid advancement of digital and AI technologies that has led to the proliferation of highly developed media, making overcommunication a pervasive issue in contemporary society. It is therefore essential that contemporary society employs technology to address the issue of sustainability, whilst also developing a deeper comprehension of the consequences of these technological advances upon the concept of sustainability. Luhmann's oversight of this impact resulted in traditional SST neglecting the problem of insufficient uncertainty stemming from overcommunication.

We consider co-creative communication to be a crucial process influencing the sustainability of social systems. Consistent with the definition in a previous study (Iba, 2016), we refer to co-creative communication as a form of social collaboration that fosters creativity within society. Today, co-creative communication is primarily enacted in the digital environment of the Internet (Zwass, 2010). As a human-specific form of social interaction, co-creative communication contributes to cultural diversity, innovation, and evolution by providing the creativity that underlies the creation and dissemination of memes (Gabora, 1997). However, co-creative communication can also bring about uncertainty in society. Encouraging creative activity usually also means facing the challenge of uncertainty, which can lead to conflicts, misunderstandings, and communication barriers (Bassett-Jones, 2005). Nevertheless, in social systems suffering from overcommunication, co-creative communication can be regarded a key process for realizing sustainability, given the new uncertainties it introduces. From this perspective, sustainability can be conceptualized as a social function inherent to co-creative communication.

Constructive approaches provide an effective method to explore the potential mechanisms of complex dynamic systems with non-reducibility (Hashimoto et al., 2008), such as communication systems involved in co-creative communication. The study of Co-Creative Communication employing Constructive approaches (CCC) holds significant value for sustainability research due to its methodological alignment and its rich repertoire of research outcomes. CCC studies focus on understanding complex phenomena through the construction and simulation of models, grounded primarily in AI methodologies. Theoretical and practical research in CCC has yielded significant research outputs in various domains.

For example, in the field of Computational Social Creativity (CSC), the methodologies developed in artificial life and computational social science are widely applied to study creativity in social communication activities (Boden, 1998; Saunders and Bown, 2015). A recent study used a computational multi-agent model with opinion dynamics to investigate the potential positive effects of “noise” in interpersonal communication on social creativity (Li et al., 2022). Another example is the field of Computational Narrative, which concerns the generation and evaluation of creative narrative works through computer modeling (Gervás, 2021). With Bayesian inference and Markov Logic Network, Chieppe et al. (2022) proposed a computational framework for modeling the creativity of short stories, such as those co-created by humans and computers. Further, creative decision-making support research has proposed methods and tools to improve the group co-creative communication process by combining methodologies developed in various fields such as AI, cognitive modeling, and inverse problems (Raikov, 2015). By investigating the factors and processes that influence co-creation in social interaction, these studies contribute to both the understanding and facilitation of co-creative communication. Given the pivotal role of co-creative communication in achieving sustainability, the findings of CCC studies have great potential to provide valuable insights into both the theoretical understanding and implementation of sustainability.

The primary challenge in applying CCC studies to the sustainability domain is the absence of a formal method to define sustainability from a communication perspective. Previous sustainability research based on AI and constructive approaches tends to view communication as a tool for resolving sustainability issues through collective decision-making (Bousquet and Page, 2004), rather than as an integral component of the definition of sustainability. Although Luhmann's SST provides a theoretical framework for defining sustainability from a communication perspective, previous research linking sustainability and SST tends to emphasize the trade-offs inherent in the development and sustainability of systems (Valentinov, 2014). Such a definition cannot provide practical advice on achieving sustainability in specific social contexts, such as those defined by the SDGs.

Therefore, the objective of this study is to develop a framework that proposes a general approach to establishing a formal definition of sustainability from a communication perspective as a basis for future applications of CCC to the study of sustainability issues. This paper presents an abstract conceptual model and an engineering approach for integrating and extending AI research findings in the field of CCC with illustrative examples. Our framework is built upon the assumption that sustainability is a property that emerges from communication in functionally independent social systems. Our approach is based on formal methods and AI technologies, including transition systems. By viewing social systems as contexts for defining specific sustainability-related issues, the proposed approach can correspond to different SDGs. By employing this framework, existing CCC research results can be extended to the study of sustainability in different contexts, offering theoretical and practical guidance for the realization of SDGs.

This paper is organized as follows. Section 2 discusses the characteristics and processes of co-creative communication. Section 3 presents the outline of the framework, highlighting the functional role of co-creative communication in the interaction between innovation and sustainability of social systems. Section 4 describes the formal method and provides illustrative examples for defining the sustainability in terms of a property of social systems. Finally, Section 5 concludes the whole study.

## 2 Co-creative communication

### 2.1 Co-creative communication process in innovation

Co-creative communication is both communication and co-creation involving multiple people. Therefore, the core of co-creative communication is the collaboration of different people. Among the different types of communication, the most important characteristic of co-creative communication is the potential for innovation. In our framework, innovation can be seen as a possible outcome of an increase in uncertainty. Co-creative communication can therefore be defined as communication that leads to a net increase in uncertainty, which is equivalent to negative information.

It should be noted that increased uncertainty means a greater possibility for new meanings to emerge, but does not necessarily lead to new meanings *per se*. In other words, what is created in co-creative communication is new alternatives for possible interpretations, which is necessary for the creation of new meanings. Thus, co-creative communication is not innovation *per se*, but a sub-process of innovation. In order to create new meanings, a decrease in uncertainty would be necessary, which requires non-creative communication. Hence, the whole innovation process involves both co-creative and non-creative communication. We suggest that effective integration of the two may be a key element in implementing innovation.

Therefore, the focus of innovation studies differs from that of sustainability studies. Although both studies address the importance of co-creative communication and uncertainty, implementing innovation is distinct from achieving sustainability. Traditionally, innovation studies prioritize reducing uncertainty, given that uncertainty is considered inherent in the innovation process and must be reduced for innovation to be achieved (Jalonen, 2012; Raikov, 2015). However, it is also necessary to consider the issue of insufficient uncertainty in sustainability research. For studies of sustainability, it is therefore important to understand the factors and processes related to both the reduction and the increase of uncertainty.

### 2.2 Distinct types of co-creative communication

**Figure 1:**
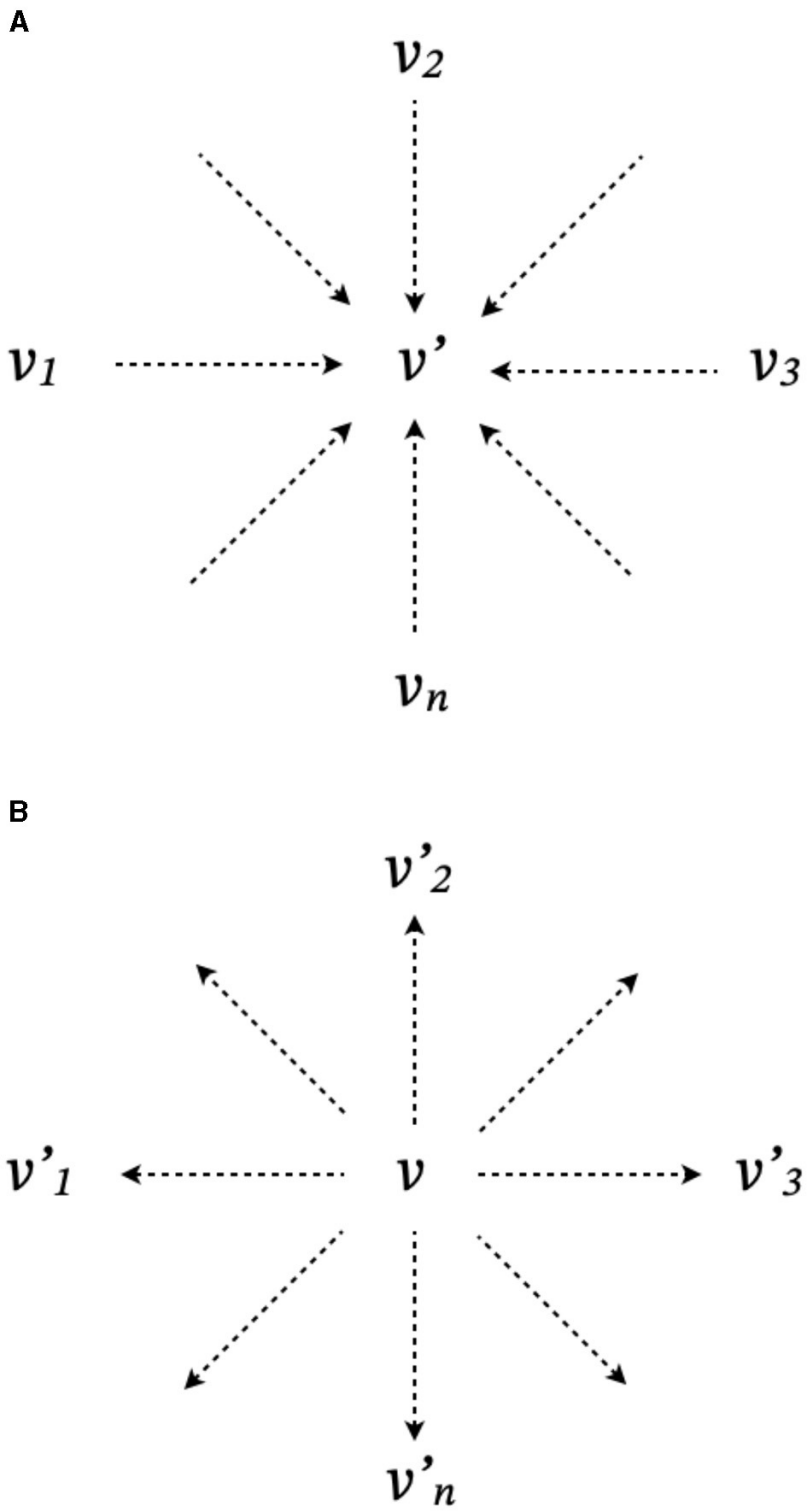
Two processes of co-creative communication: **(A)** *synthesis* and **(B)** *destruction*. Dotted arrows indicate potential transformation from existing meaning (*v*) to new alternatives (*v*′).

Co-creative communication increases uncertainty by generating new alternatives of meaning. In practice, this can be achieved through more than one mechanism. First, new alternatives can be created through a synthesis process, which is proposed in Nonaka's theory of knowledge creation (Nonaka, 1994; Nonaka and Toyama, 2005). The synthesis process for creating a new alternative interpretation is illustrated in Figure 1A. This process takes different existing meanings (*v*_1_, *v*_2_, …, *v*_*n*_) for the inputs. The innovative output (*v*′) is unknown prior to communication, but in the same domain as the input.

Synthetic co-creative communication represents a key challenge in the field of innovation support research. This field of research focuses on the development of techniques and frameworks for supporting innovation processes. One of the primary topics of interest is the facilitation of effective decision-making and collective insight generation. For example, Raikov (2015) proposed a method and specific software implementation that accelerates creative decision-making within groups of experts in digital environments through convergent information structuring. This line of research supports two critical processes in innovation: identifying effective solutions and achieving consensus on the optimal solution. The former is related to the synthetic co-creative communication, while the latter is related to the non-creative communication.

On the other hand, new alternatives can also be the result of a destruction process that Schumpeter (1976) called *Creative Destruction*. The destruction process for the creation of new alternative interpretations is illustrated in Figure 1B. This process takes an existing meaning (*v*) as inputs and produces one or more innovative alternatives (*v*_1_, *v*_2_, …, *v*_*n*_). One of the most obvious differences from the process of synthesis is that the process of destruction has only one input, rather than a number of inputs. Another important difference is that the output of the destruction process is not constrained by the domain of input, which allows for the generation of a diverse range of products. This feature is consistent with a Schumpeterian view of service innovation (Drejer, 2004).

Destructive co-creative communication plays a significant role in co-creation activities that utilize AI techniques. For instance, Epstein et al. (2022) investigated methods for leveraging text-to-image generation AI models to support idea visualization, thereby facilitating creative collective conversations (a form of brainstorming) within communities. Their findings revealed that high-variance, unpredictable lo-fi generative AI was more conducive to generating novel insights and possessed a wide range of potential applications. This line of research encompasses support for both co-creative communication and non-creative communication. The former is a destruction process that derives new ideas based on visualization results that are inconsistent with original thoughts. The latter drives conversation and communication among communities.

Both synthesis and destruction processes can increase uncertainty. The main difference is the number of inputs and the range of interpretation. Due to the distinction between input and output, the two processes are applicable in different contexts. Both processes can be involved when implementing innovation through co-creative communication.

## 3 Co-creative communication as a key to achieving sustainability

**Figure 2:**
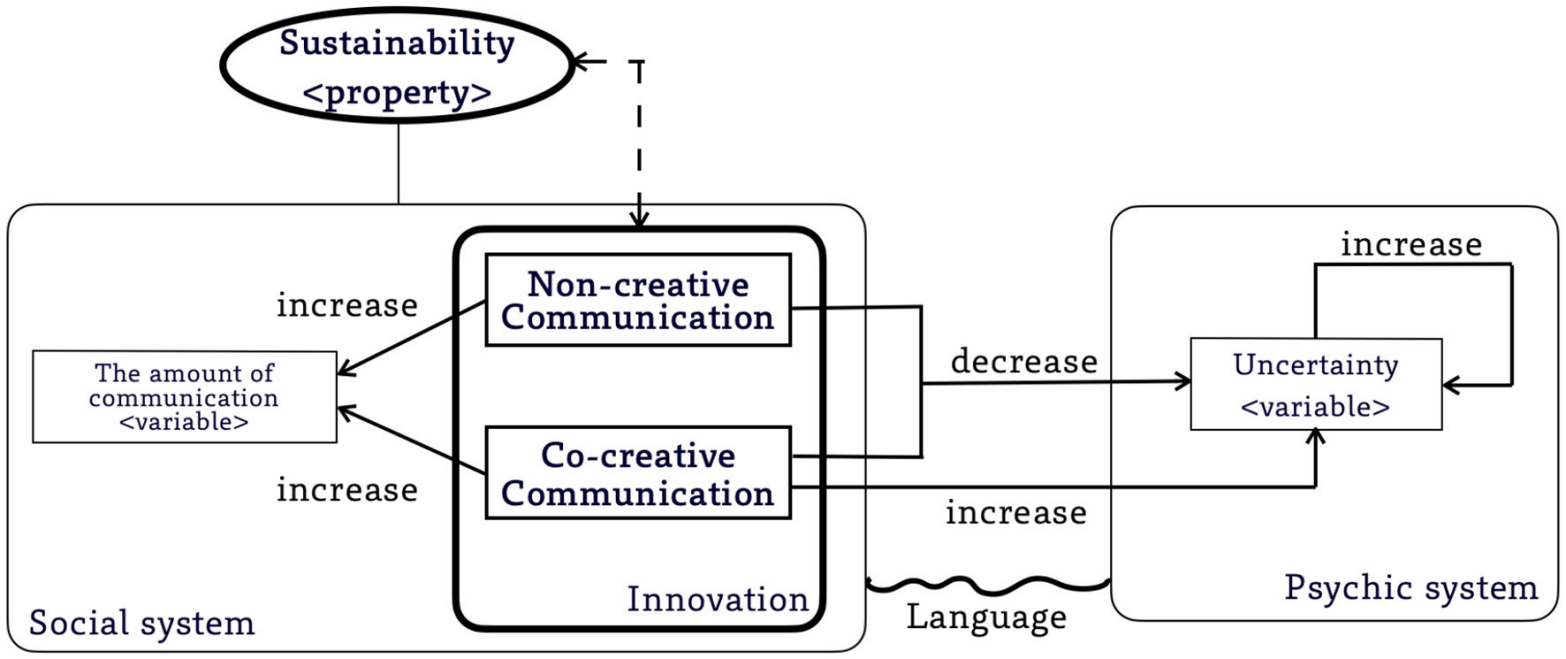
The interaction of *Innovation* and *Sustainability* and a functional role of *co-creative communication*. Arrows: the direction of effects. Solid arrows indicate direct effects. Dotted arrows indicate indirect effects. Wavy line: structural coupling between systems.

To link a CCC study to investigate sustainability problem, we need to consider an ecosystem composed of psychic systems and social systems. Figure 2 shows the whole picture of our framework.

Consistent with the view of social systems theory (Iba, 2016), the psychic systems are structurally coupled to social systems through language. In the social systems, the element is communication, which includes both co-creative and non-creative communication. Communication produces more communication, while consuming the uncertainty in the psychic systems. The uncertainty can be recovered naturally, at a rate that depends on the resilience of the psychic systems.

*Sustainability* is defined as a property of social systems that emerges from communication. To determine sustainability, we focus on two critical variables in social systems and psychic systems. In social systems, we focus on *The amount of communication*, denoted as *N*_*c*_. In psychic systems, we focus on *Uncertainty*, denoted as *H*. The innovation activity consists of two sub-processes, co-creative communication and non-creative communication, both of which consume uncertainty (*H*) and lead to an increase in the amount of communication (*N*_*c*_). Meanwhile, co-creative communication produces more uncertainty than it consumes, resulting in a net increase in uncertainty, i.e., a positive Δ*H*.

This framework can correspond to many forms of innovative activities. In some innovative activities, both co-creative and non-creative communication are given equal weight. Brainstorming places more emphasis on co-creative communication, which facilitates the realization of sustainability but is not conducive to the realization of system functionality. Convergent innovation, where consensus is required, places more emphasis on non-creative communication, which increases the efficiency of communication but is not conducive to the realization of sustainability. Thus, there is an interaction between sustainability and innovation: the realizability of sustainability determines the ideal form of innovation activities, and the practice of innovation activities affects the realizability of sustainability.

Our framework assumes that co-creative communication, a sub-process of innovation, serves a social function by enhancing the capacity of social systems to achieve sustainability. Co-creative communication offers an active approach to generating productive uncertainty. This uncertainty can be addressed through further communication, ultimately strengthening the ability of social systems to maintain functionality even in the face of significantly increased communication efficiency.

Real-world scenarios corresponding to this framework can be demonstrated in the context of scientific research. For science as social systems, institutional frameworks can foster innovation in underdeveloped systems but, conversely, may hinder sustainability in overdeveloped ones. Specifically, for an underdeveloped science system with high uncertainty, institutional development (e.g., establishing consensus on evaluating research outcomes) can significantly reduce uncertainty and promote scientific innovation. Conversely, an overdeveloped institutional framework can lead to sustainability challenges for scientific research due to rigidity and a lack of adaptability. The latter scenario aligns with the funding issues for basic and translational research raised in the recent trend toward a decentralized science (DeSci) paradigm (Strauss, 2023a,b). The DeSci paradigm is based on the development of Web3.0 and decentralized autonomous organizations (DAOs) (Ding et al., 2022). As a co-creative communication platform, DeSci can introduce productive uncertainty into an overdeveloped science system by encouraging diverse perspectives and open communication. Hence, DeSci directly corresponds to the functional link between co-creative communication and sustainability within our framework.

This framework leverages AI research on co-creative communication to advance sustainability theory and practice. For instance, the framework allows us to distinguish theoretical and constructive studies in Computational Social Creativity and Computational Narrative, which utilize AI methods like Multi-Agent Simulation and Bayesian modeling. While they primarily focus on the effects of innovation on the amount of communication (*N*_*c*_) and uncertainty (*H*), these studies also differ in specific factors. For example, Gervás (2021) reviewed computational narrative models that explored the impact of creative narratives on *H* rather than *N*_*c*_. Li et al. (2022) used Multi-Agent Simulation to examine the impact of individual communication on society arising from co-creative communication, not the entire innovation process. On the other hand, AI innovation research emphasizes using AI technologies, including generative AI, to facilitate and support innovation processes that encompass both co-creative and non-creative communication. These studies can be categorized according to their focus, such as the studies focusing on co-creative communication [e.g., the use of text-to-image models to support open discussion in communities (Epstein et al., 2022)], vs. non-creative communication [e.g., convergent approach to support networked group decision-making (Raikov, 2018)]. Our framework also reveals a recent trend: a shift in focus from productive uncertainty (*H*) to societal effects (*N*_*c*_), in the context of the widespread adoption of generative AI (Lamb and Brown, 2023). Differentiating the findings of these studies is important because the factors they focused on play different roles in sustainability research.

In order to adapt these findings to explore sustainability issues, we need to develop a formal definition of the *sustainability* property in the corresponding research context. The next section describes this approach.

## 4 Sustainability of social systems

This section presents a formal method to define sustainability in the context of social systems. The following subsections will firstly present the formal method for describing the behavior of social systems, before going on to illustrate this approach for a typical social system. Finally, we will outline the method for defining sustainability as a property using formal logic and present illustrative examples.

### 4.1 Transition systems

Conventionally, sustainability research views sustainability as a state or a process to achieve this state (Mensah, 2019). However, due to the broad scope of SDGs, it is very unlikely that there exists a social state or a social transition that can be labeled as “sustainability”. Moreover, even if such an ideal state or transition can be defined, sticking with it would be too difficult because of the dynamic interaction between social and psychic systems. Instead, sustainability should be viewed as a desired property of social systems that is manifested in its dynamic and evolving behavior.

In order to apply CCC studies to sustainability research, it is first necessary to establish a formal definition of sustainability for a social system. This can be achieved by employing formal methods to firstly describe the behavior of the system and then represent the desired sustainability property. The behavior of social systems can be described by using *transition systems*. Transition systems are abstract systems composed of discrete *states* and *transitions* between them (Keller, 1976). Conventionally, a transition system is defined as (*Q*, →), where *Q* is a set of states and → is a set of transitions representing the binary relation on *Q*. The notion of transition systems provides a fundamental tool for modeling dynamic systems with interacting processes. Based on such modeling, we can verify the properties of a system or compare different systems by means of their logical equivalences (Arnold, 1993).

In transition systems, a *property* of a system refers to certain characteristics of the transition path of that system. These *properties* of the transition systems can be automatically verified by mathematically based formal methods (Clarke and Wing, 1996). For that purpose, the *properties* need to be expressed by using formal logic, which is a formal language of mathematical logic (Arnold, 1993). Mathematical logic forms the theoretical foundations of AI, especially in the field of knowledge representation and logical inference (McCarthy, 1988). Specifically, we use Computation Tree Logic (CTL) (Sistla, 1994), a type of formal logic, to define sustainability. CTL consists of atomic propositions and Boolean connectives. It is an efficient formalism for describing all possible transitions between states in a transition system.

Transition systems and CTL have been widely used in AI research, including inference systems and natural language processing (NLP). For example, Bench-Capon (2015) applied transition systems and CTL to multi-agent systems for exploring the designing and reasoning about norms. Transition systems have also been actively used in NLP studies as a basis for designing text parsers (Fernández-González and Gómez-Rodríguez, 2020).

In this work, transition systems are used to model social systems, and CTL is used to formally represent *sustainability* as a verifiable property. This approach allows for the extension of AI research findings to sustainability research. The specific definitions of *sustainability property* may vary depending on the specific CCC models and issues of interest. However, in order to establish formal definitions, there are common steps that must be followed. First, it is necessary to identify the *states* and *transitions* of the related social systems. Secondly, *sustainability property* needs to be represented using formal logic such as CTL. In the following subsections, basic examples are provided to demonstrate how to construct the *sustainability property* in typical scenarios.

### 4.2 Sustainability related states and transitions

Usually, CCC studies employ computational/symbolic models of interpersonal communication. Applying such models to sustainability research requires that the researcher first specify the relevant social system, which corresponds to the model, and then identify and assess its potential states and transitions. For a given social system, the states can be specified according to the researcher's concerns. Typically, in most cases, two different dimensions can be used to identify the states that are relevant to sustainability: a growth/degrowth (*G-DeG*) dimension and a sustainable/unsustainable (*S-UnS*) dimension.

The G-DeG dimension corresponds to the internal states of social systems. For a social system, a growth means an increase in the internal complexity (Valentinov, 2014). The growth/degrowth issues are not relevant to the communicating agents, although they are critical to the social system. Meanwhile, the S-UnS dimension corresponds to the impacts of psychic systems. A social system is sustainable as long as communication is maintained. Therefore, it is the psychic system, which is outside the social system, that determines whether to communicate or not.

**Figure 3:**
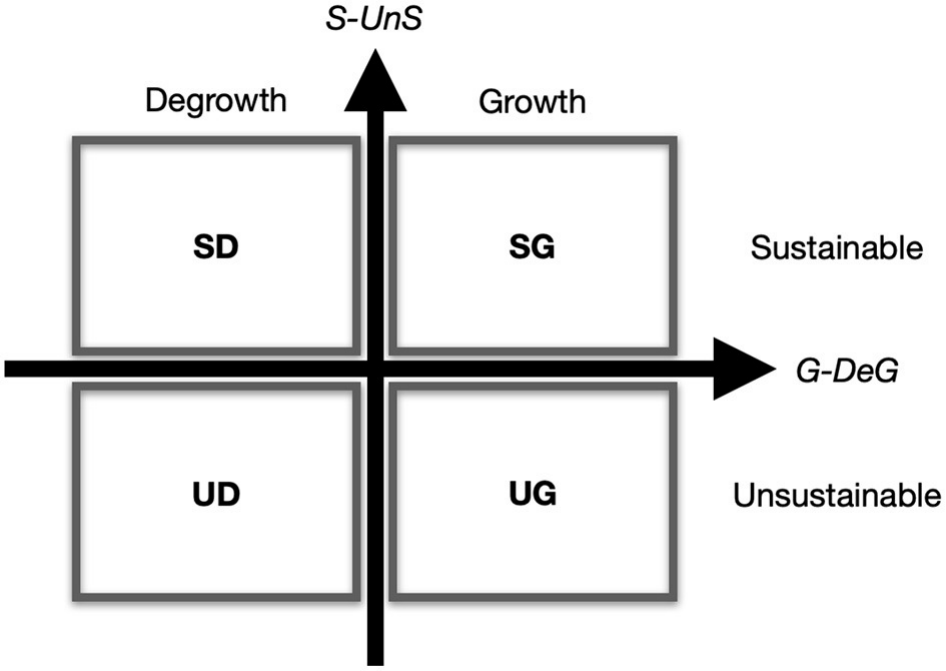
Potential states related to the sustainability of typical social systems. Four states are differentiated in two dimensions: sustainable degrowth (*SD*), sustainable growth (*SG*), unsustainable degrowth (*UD*), and unsustainable growth (*UG*). Horizontal dimension: growth/degrowth (*G-DeG*). Vertical dimension: sustainable/unsustainable (*S-UnS*).

Accordingly, four sustainability related states can be distinguished: sustainable growth (*SG*), unsustainable growth (*UG*), sustainable degrowth (*SD*), and unsustainable degrowth (*UD*). These states, arranged along the two dimensions, are shown in Figure 3.

We can then measure the social system by designing indicators for each of the two dimensions. In the dimension G-DeG, the state of the system can be measured by changes in the amount of communication (Δ*N*_*c*_). This indicator is independent of communicating agents and can be seen as indirectly reflecting the social system's internal complexity.

For the dimension S-UnS, we can measure the system states by changes in the uncertainty (Δ*H*). This indicator can be calculated as the sum of all communicating agents. A continuous decline in uncertainty suggests that the system may suffer from overcommunication, a scenario involving overdevelopment.

**Figure 4:**
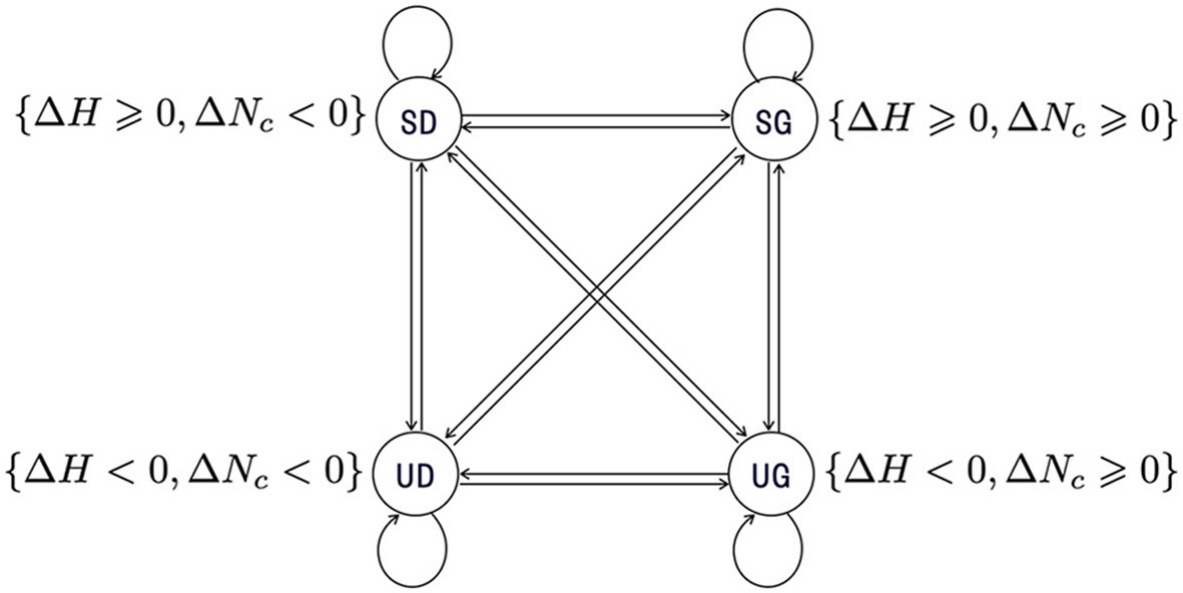
A transition system consisting of four sustainability related states, each associated with a descriptive label. The transition relationships between states are modeled as fully connected, in accordance with the intrinsic stochastic characteristics inherent to social transitions. Circles: the states. Arrows: the transitions from a start state to an end state.

Next, we need to specify the transitions between these states. However, in most cases, state transitions in social systems are intrinsically stochastic, and it is very difficult to specify precisely the transition paths of the system. For simplicity, we can model the transition relationships between states as fully connected. Accordingly, a typical transition system of social systems with respect to the states related to sustainability can be constructed as in the Figure 4.

### 4.3 Sustainability as a property of a social system

Based on the constructed transition system for CCC models, we can then define sustainability as a *property* of social systems using formal logic such as CTL. Depending on the context, the specific definition of *sustainability* may vary. As examples, the following considers the definition of *sustainability property* in two distinct contexts related to the economy system: the context of regional economic revitalization, where sustainability is defined in a weaker sense, and the context of the tourism resources preservation, where the definition is in a stronger sense.

The CCC models can be applied to sustainability research in the context of regional economic revitalization. In this context, the primary goal is to achieve and sustain local economic growth. Consequently, related research tends to focus on balanced development through the fostering of innovation systems (Yin et al., 2022), that is, how to move away from being both unsustainable and economic degrowth through innovation. This issue can be explored through the application of CCC models that are concerned with the social interactions within the local economy system.

**Figure 5:**
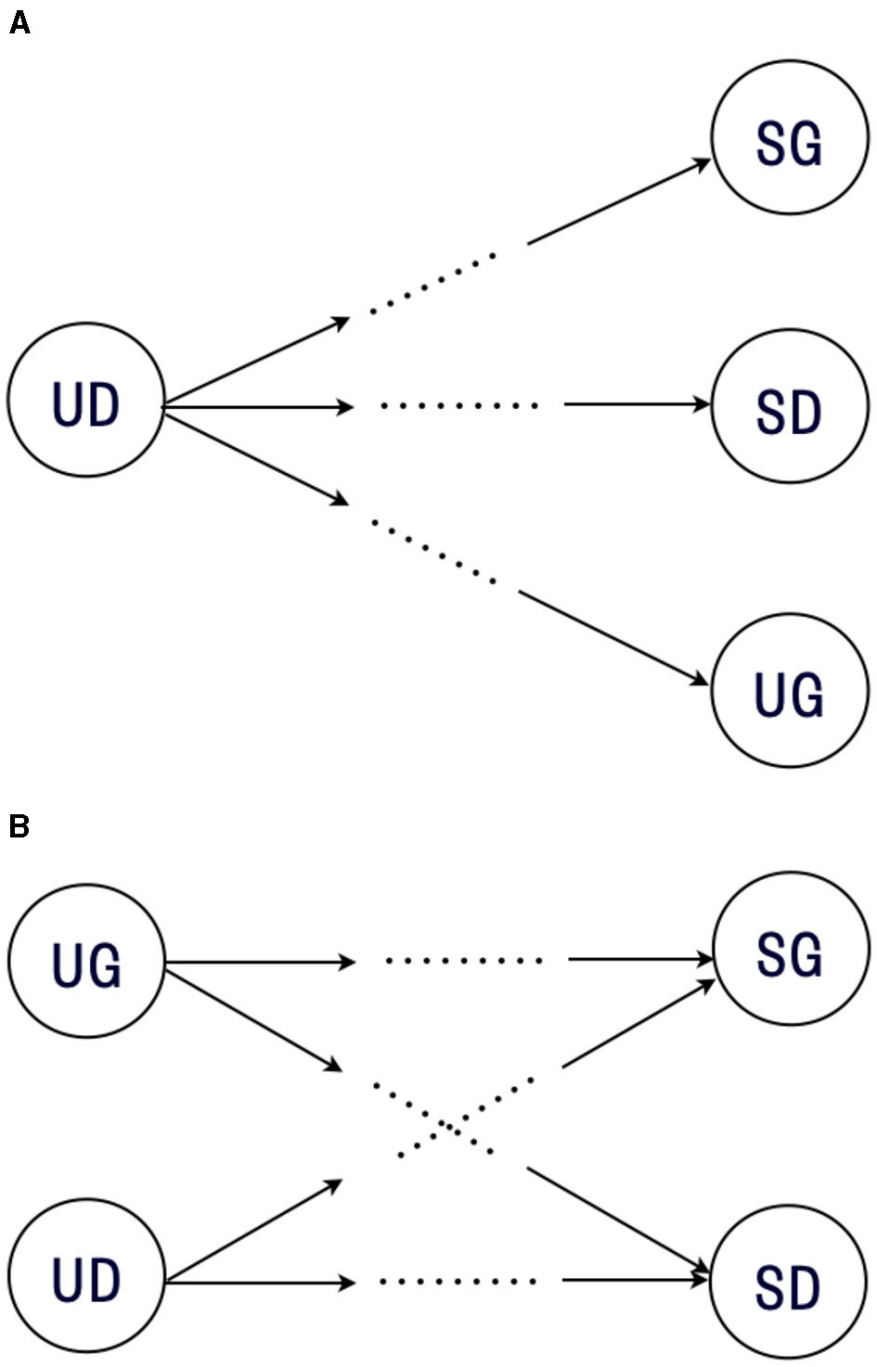
Transition paths of **(A)** *weak sustainability* and **(B)** *strong sustainability*. Circles: the states. Arrows: the transitions from a start state to an end state. Dotted lines indicate omitted transitions in the path. **(A)** Corresponding to a social system that can always jump out of the unsustainable degrowth (*UD*) state after a series of transitions. **(B)** Corresponding to a social system that can always move away from unsustainable (*UG* & *UD*) states after a series of transitions.

For example, Zhang (2003) investigated the regional economic growth achieved by Schumpeterian entrepreneurship, which can be considered as an instance of social communication between local agents. By employing agent-based modeling, the model simulates entrepreneurial activities on a blank landscape and shows the spontaneous formation of industrial clusters, such as those in Silicon Valley. This model can be applied to sustainability research by defining the sustainability property for the local economy system. Following the transition systems defined previously (see Figure 4), a system that can meet sustainability should have transition paths shown in Figure 5A. The *sustainability property* defined here may be referred to as *weak sustainability*, which is satisfied if a system can always jump out of the unsustainable degrowth (*UD*) state after a series of transitions.

The above definition of sustainability can be formally represented as a *liveness* property for transition systems, which expresses “eventually something (good) must happen” (Lamport, 1977; Kindler, 1994). A liveness property that conforms to CTL consists of *path quantifiers* and *temporal operators*. The path quantifiers are **A** (for all paths) and **E** (there exists a path). The temporal operators are **X** (next time), **F** (eventually), **G** (always), **U** (until), and **R** (release). Hence, *weak sustainability* can be formally defined as:


$$\text{AG}\left(\left(\Delta H<0\wedge \Delta N_{c}<0\right)\to \text{AF}\left(\Delta H\geq 0\vee \Delta N_{c}\geq 0\right)\right)$$

which can be automatically verified for each run of the computer simulation with the model.

In contrast, the sustainability research in the context of the preservation of tourism resources would have a primary goal to resources preservation for future generations (Angelevska-Najdeska and Rakicevik, 2012). In this context, it is of greater importance to identify the factors that lead to a sustainable state. Consequently, the sustainability of a local economy system should have transition paths shown in Figure 5B, which may be referred to as *strong sustainability*.

The *strong sustainability* is satisfied if a system can always move away from any unsustainable states (whether that be growth or degrowth) after a series of transitions. This property can be defined in CTL as:


AG(ΔH<0→AF(ΔH≥0))

which can be verified for CCC models focusing on the social interactions between various agents, including tourists and local residents.

It should be noted that not all sustainability positions can be defined in terms of a dichotomy between strong and weak. An example of this can be seen in another proposal for sustainable tourism (Vu et al., 2020), which emphasizes the concepts of hand-in-hand development and resources protection. This can also be defined as a *liveness* property with CTL. The flexibility in definition permits the incorporation of diverse CCC research findings into the domain of sustainability research.

## 5 Conclusion

In conclusion, this paper presents a framework that reconciles the studies employing Constructive approaches to Co-creative Communication (CCC) and sustainability research from a communication perspective. The framework delineates the essence of sustainability issues within social functional systems, identifying them as problems of overdevelopment stemming from a lack of productive uncertainty. It also highlights the interplay between sustainability and innovation processes, where co-creative communication in innovation activities exhibits social functions for facilitating the realization of sustainability by introducing new uncertainties for future development.

While the proposed framework is not a fully validated model, it provides a solid foundation for future research endeavors. As CCC research on sustainability progresses, empirical studies can be conducted to validate and refine the framework's components and mechanisms.

This framework not only provides a formalized definition and illustrative examples of sustainability issues, but also demonstrates its adaptability across diverse contexts, accommodating the unique characteristics of various sustainability problems. The framework serves as a tool for applying computational creativity research based on AI technologies to understand and address sustainability challenges. This work thus enriches our understanding of the mechanisms underlying sustainability issues and facilitates their practical applications.

Furthermore, viewing fostering sustainability as a social function of co-creative communication opens avenues for future discussions on the adaptive evolution of co-creative communication practices. This paves the way for exploring the evolving dynamics of co-creative communication in societal contexts.

## Data availability statement

The original contributions presented in the study are included in the article/supplementary material, further inquiries can be directed to the corresponding author.

## Author contributions

GL contributed to the writing of the manuscript and funding acquisition for this work. XG critically reviewed the manuscript and assisted in its preparation. All authors listed have made a substantial, direct, and intellectual contribution to the work and approved it for publication.
